# The effects of antibiotic exposure on asthma in children with atopic dermatitis

**DOI:** 10.1038/s41598-021-87981-7

**Published:** 2021-04-19

**Authors:** I-Lun Chen, Ming-Kai Tsai, Hao-Wei Chung, Hui-Min Hsieh, Yu-Ting Huang, Yi-Ching Lin, Chih-Hsing Hung

**Affiliations:** 1 Department of Pediatrics, Kaohsiung Chang Gung Memorial Hospital, Chang Gung University, College of Medicine, Kaohsiung, Taiwan; 2 Division of Nephrology, Department of Internal Medicine, Kaohsiung Armed Forces General Hospital, Kaohsiung, Taiwan; 3 Department of Pediatrics, Kaohsiung Medical University Hospital, Kaohsiung Medical University, Kaohsiung, Taiwan; 4 Department of Public Health, Kaohsiung Medical University, Kaohsiung, Taiwan; 5 Department of Medical Research, Kaohsiung Medical University Hospital, Kaohsiung Medical University, Kaohsiung, Taiwan; 6 Department of Community Medicine, Kaohsiung Medical University Hospital, Kaohsiung, Taiwan; 7 Center for Big Data Research, Kaohsiung Medical University, Kaohsiung, Taiwan; 8 Division of Medical Statistics and Bioinformatics, Department of Medical Research, Kaohsiung Medical University Hospital, Kaohsiung Medical University, Kaohsiung, Taiwan; 9 Department of Laboratory Medicine, Kaohsiung Medical University Hospital, Kaohsiung Medical University, Kaohsiung, Taiwan; 10 Doctoral Degree Program of Toxicology, College of Pharmacy, Kaohsiung Medical University, Kaohsiung, Taiwan; 11 Department of Laboratory Medicine, School of Medicine, College of Medicine, Kaohsiung Medical University, Kaohsiung, Taiwan; 12 Research Center for Environmental Medicine, Kaohsiung Medical University, Kaohsiung, Taiwan; 13 Department of Pediatrics, Faculty of Pediatrics, College of Medicine, Kaohsiung Medical University, Kaohsiung, Taiwan; 14 Graduate Institute of Medicine, College of Medicine, Kaohsiung Medical University, Kaohsiung, Taiwan; 15 Department of Pediatrics, Kaohsiung Municipal Siaogang Hospital, Kaohsiung, Taiwan

**Keywords:** Paediatric research, Allergy, Asthma, Atopic dermatitis

## Abstract

Early-life antibiotic use is associated with allergic diseases. The risk factors for the progression from atopic dermatitis (AD) to asthma or allergic rhinitis are still unknown. We aimed to investigate the association between exposure to different antibiotics and the risk of new-onset asthma in children with AD. By using the Longitudinal Health Insurance Database 2005, we selected AD patients less than 6 years old identified by ICD-9-CM code 691.8. The case group was defined as those having new-onset asthma, and the control group was defined as those without an asthma history. Information on antibiotic exposure in the 5 years prior to the index date was collected from drug prescription records. We estimated the adjusted odds ratio by using conditional logistic regression, adjusted for age, sex, index year, other potential risk factors and antibiotics. Antibiotic exposure was associated with the development of asthma in patients with AD (aOR = 3.68, 95% CI 2.13–6.36), particularly for patients less than 5 years old (aOR = 4.14, 95% CI 2.24–7.64) (p for trend < 0.001), even though lower cumulative antibiotic defined daily doses (DDDs) were associated with new-onset asthma occurrence. Antibiotic exposure, especially macrolide exposure, is associated with an increased risk of asthma in patients with AD.

## Introduction

The pathogenesis of atopic dermatitis (AD), the most common pruritic inflammatory skin disease in children, is characterized by multiple and complex factors. The age of onset, severity of the illness, gene expression and trigger factors could be related to different mechanisms^1^. Patients with refractory AD are characterized by early-onset AD, age younger than 2 years old, and severe long-term disease associated with allergic asthma and/or food allergy.

The “atopic march” is a term used to describe the allergic development from AD in infancy to subsequent allergic rhinitis (AR) and allergic asthma (AA) in preschool age^2^. An animal model study showed that the progression from AD to AA may occur because of skin barrier dysfunction and abnormal interactions between the epithelium and microorganisms^3,4^. 

Antibiotics are frequently prescribed for upper and lower respiratory tract infections in children, and global antibiotic consumption is also gradually increasing^5,6^. The associations of prenatal and postnatal exposure to antibiotics with the later development of allergic diseases in children have been reported in several studies^7–10^. A twin study controlling for genetic and environmental factors showed that early-life antibiotic use increased the risk of the development of asthma but not eczema^11^. Antibiotic use during infancy may alter the intestinal microbiota and immune development, which is associated with an increased risk of childhood asthma. The gut microbiota plays an important role in regulating the immune system of CD4+ T cells to protect against immunoglobulin E induction^12^. However, the overuse of antibiotics changes the distribution of the gut microbiota and leads to less microbial diversity, which needs to recover for a long time from exposure to antibiotics^13–15^. Thus, lifetime antibiotic exposure changes the construction and variety of the gut microbiome, which might be related to the development of allergic diseases^16^.

Our previous study also concluded that, in children, 5 preceding years of antibiotic exposure, predominantly to amoxicillin and macrolides, was associated with the risk of asthma development in AR and in bronchiolitis^17,18^. However, the risk of the development of new-onset asthma in AD patients due to early-life antibiotic exposure is still unknown. The first aim of this study was to prospectively assess whether previous antibiotic use is a risk factor for the new diagnosis of asthma in children with AD. The second aim was to investigate the effect of antibiotic dosage and whether the subtype of antibiotic used is critical for asthma development in patients with AD.

## Results

### Demographic data of the study subjects

After 1:3 individual matching, there were 1251 AD patients with new-onset asthma (case group) and 3753 patients without asthma (control group) in this study. The demographic characteristics of the case and control groups at baseline are shown in Table 1. A total of 63.9% of patients were less than 5 years old, and 58.2% were male. Enrolled patient age, sex, comorbidities, and comedications were not different between the case and control groups (Table 1).

**Table 1:** Demographic characteristics between cases and controls in children with atopic dermatitis.

	Before matching			After matching		
	Case group	Control group	P-value*	Case group	Control group	P-value*
	(N, %)	(N, %)		(N, %)	(N, %)	
N	1315	6491		1251	3753	
**Gender (N, %)**						
Female	554 (42.1%)	3357 (51.7%)	< 0.0001	523 (41.8%)	1569 (41.8%)	1.000
Male	761 (57.9%)	3134 (48.3%)		728 (58.2%)	2184 (58.2%)	
**Age group (N, %)**						
< 5	846 (64.3%)	3704 (57.1%)	< 0.0001	800 (63.9%)	2400 (63.9%)	1.000
5–9	436 (33.2%)	2475 (38.1%)		421 (33.7%)	1263 (33.7%)	
> 10	33 (2.5%)	312 (4.8%)		30 (2.4%)	90 (2.4%)	
**Residential area (N, %)**						
Taipei area	538 (40.9%)	2278 (35.1%)	< 0.0001	506 (40.4%)	1585 (42.2%)	0.0016
Northern area	248 (18.9%)	727 (11.2%)		239 (19.1%)	544 (14.5%)	
Central area	173 (13.2%)	1046 (16.1%)		161 (12.9%)	586 (15.6%)	
Southern area	196 (14.9%)	1284 (19.8%)		190 (15.2%)	604 (16.1%)	
Kao-Ping area	147 (11.2%)	1042 (16.1%)		144 (11.5%)	399 (10.6%)	
East area	13 (1.0%)	114 (1.8%)		11 (.9%)	36 (.9%)	
**Comorbidity (N, %)**						
Allergic rhinitis	469 (35.7%)	785 (12.1%)	< 0.0001	405 (32.4%)	1173 (31.3%)	0.4607
Chronic rhinitis	159 (12.1%)	484 (7.5%)	< 0.0001	142 (11.4%)	380 (10.1%)	0.2194
Acute sinusitis	811 (61.7%)	2658 (40.9%)	< 0.0001	757 (60.5%)	2351 (62.6%)	0.1811
Bronchiolitis	183 (13.9%)	287 (4.4%)	< 0.0001	139 (11.1%)	427 (11.4%)	0.7966

*Chi-square test was used to compare patients’ characteristics between case and controls in children with atopic dermatitis and p values < 0.05 indicating statistically significant difference.

### The relationship between antibiotic subtype and cumulative defined daily doses (DDDs) and the risk of asthma

The association between early antibiotic exposure and asthma development in patients with AD was analyzed. In the present study, antibiotic exposure was associated with the development of asthma in patients with AD (aOR = 3.68, 95% CI 2.13–6.36; Table 2), in particular for patients age less than 5 years old (aOR = 4.14, 95% CI 2.24–7.64; Table 2). To assess which specific antibiotic subtypes were associated with an increased risk of asthma development, further analysis was performed (Appendix eTable 1). Interestingly, exposure to the antibiotics azithromycin, clarithromycin, cefazolin sodium, amoxicillin, and cephradine in AD patients had higher odds ratios for new-onset asthma diagnosis at a younger age (< 5 years old), while only AD children with azithromycin, clarithromycin, and amoxicillin exposure had higher risks for asthma diagnosis at older time points (> 5 years old).

**Table 2:** Antibiotic exposure and the risk of ages of asthma development in children with atopic dermatitis.

	Case group		Control group		Adjusted model^a^		
	N (exposure/total)	%	N (exposure/total)	%	aOR	(95%CI)	P-value
Over all	1236/1251	98.8	3599/3753	95.9	3.68	2.13–6.36	< 0.0001
< 5 years	788/800	98.5	2265/2400	94.4	4.14	2.24–7.64	< 0.0001
> 5 years	448/451	99.3	1334/1353	98.7	2.12	0.62–7.24	0.2303

aOR: adjusted odds ratios; CI: confidence intervals.

P values < 0.05 were statistically significant.

^a^Conditional logistic regression models were used and adjusted gender, age group, residential area, and comorbidities covariates listed in the Table 1.

**Figure 1:**
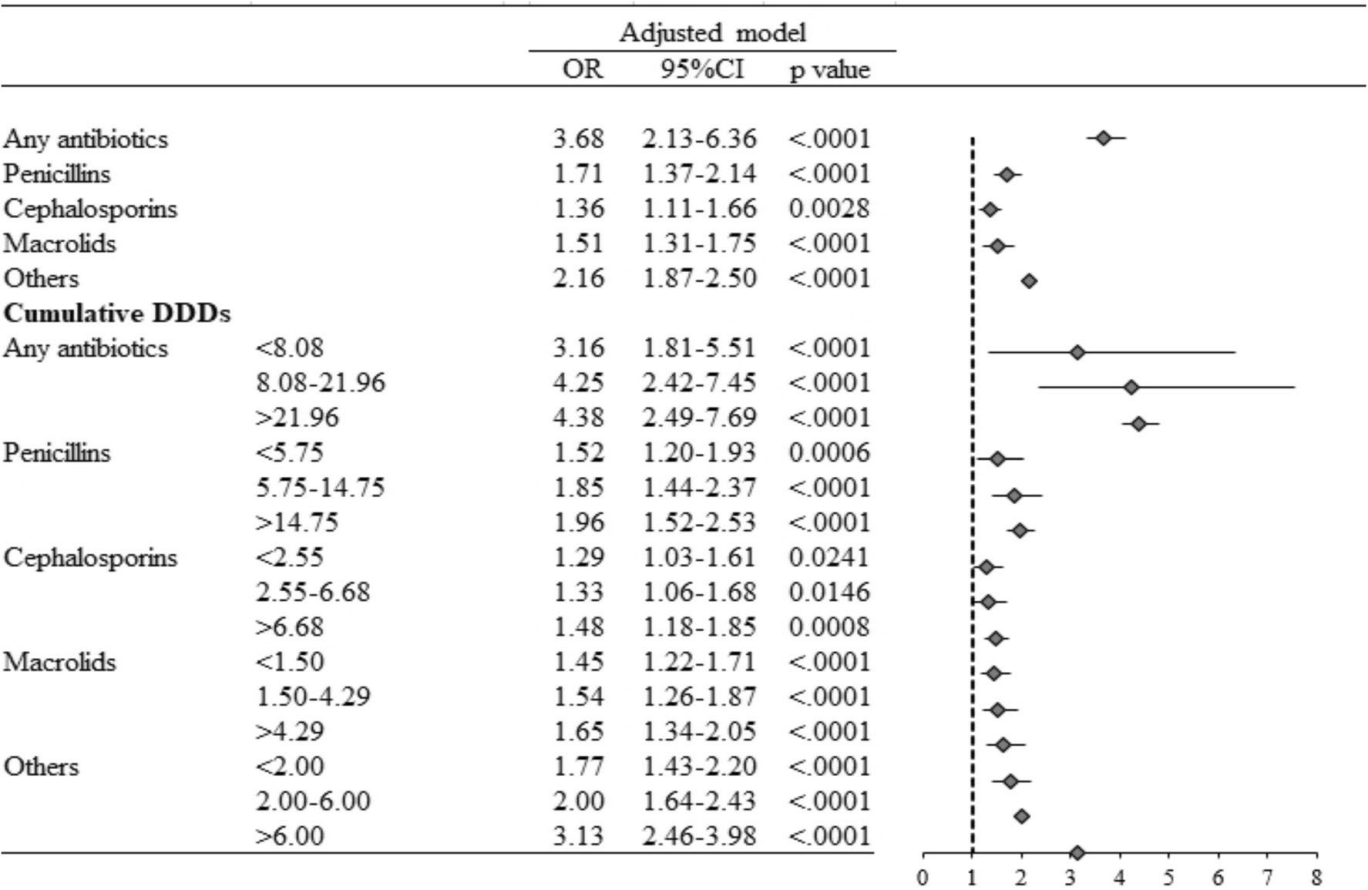
Forest plot of summarized results for cumulative DDDs of antibiotics within the 5 years preceding the index date and the risk of asthma development in children with atopic dermatitis.

The association between the cumulative DDDs of antibiotics and asthma development was analyzed in AD patients less than 5 years old (Fig. 1; Appendix eTable 2). Among 1251 AD children with new-onset asthma, 1236 (98.8%) received antibiotic treatment within 5 years prior to the index date, and 1138 (91%), 1094 (87.5%), 799 (63.9%), and 559 (44.7%) received at least one penicillin, cephalosporin, macrolide or other type of antibiotic prescription, respectively (Appendix eTable 2). As shown in Fig. 1, the overall exposure to antibiotics had a dose-dependent relationship with new-onset asthma (Fig. 1). The highest cumulative DDDs of penicillins (> 14.75), cephalosporins (> 6.68), macrolides (> 4.29) and others (> 6.00) were associated with the development of new-onset asthma at age < 5 years old (Fig. 1; Appendix eTable 2). Moreover, the use of macrolides had a higher OR, not only in the age less than 5 years group (adjusted OR = 3.80, 95% CI 2.80–5.18) but also in the age greater than 5 years group (adjusted OR = 3.04, 95% CI 2.21–4.18) (Appendix eTable 1).

## Discussion

This study revealed that AD patients with more antibiotic use would have a higher risk of asthma development. After age stratification, the use of azithromycin was associated with the highest risk of new-onset asthma overall and in asthma patients less than 5 years old. Only three cases without antibiotic exposure in this study were diagnosed asthma more than 5 years old, however, the association between antibiotic exposure and the development of asthma in patients aged more than 5 years is not significant.

Worldwide, sensitization to allergens is found in almost 20–40% of the population^19^. Allergic diseases such as allergic rhinitis, asthma, and atopic dermatitis are common chronic inflammatory diseases in the world, resulting in great economic burden in terms of health-care costs. In the past several decades, the prevalence of allergic diseases has increased rapidly worldwide with the overuse of antibiotics^15^.

The gut microbiota is important in human health, and the intestinal ecosystem of commensal bacteria contributes to the development of immunological balance. Antibiotic exposure, which kills commensal bacteria, will destroy the intestinal epithelial barrier and may be one of important factors to exacerbate the “atopic march”^4,14,15^. According to the hygiene hypothesis, allergic diseases have increased in recent decades due to low exposures to a wide array of infections, which have the ability to modulate the human immune system^20,21^. Early exposure to bacteria and parasites during childhood are key factors in preventing allergies and autoimmune diseases^22^. Therefore, antibiotic exposure may also contribute to the risk of developing asthma in AD patients. In the other hand, probiotics such as strains of lactobacilli and bifidobacteria are the most commonly applied for the prevention of allergic disease. A large meta-analysis suggested that probiotics might prevent AD but not other allergic diseases^23^. Our study suggested that antibiotic use, especially the use of macrolides, in AD patients should be cautioned due to the relationship with the development asthma in young children.

The treatment of AD includes pruritus control, skin hydration and education. In addition, *Staphylococcus aureus* colonization, particularly methicillin-resistant *S. aureus* (MRSA), commonly occurs in patients with AD. The colonization of *S. aureus* has been reported in AD skin more than in healthy skin (60–100% vs. 5–30%)^24–26^ and contributes to the exacerbation of AD. Thus, topical or systemic antibiotics are unavoidably used in patients with AD to treat or prevent AD complications, such as cellulitis or other skin infections. Among 1251 AD children with new-onset asthma, 1236 (98.8%) received an antibiotic prescription within 5 years prior to the index date, 1138 (91%) received at least one penicillin prescription, 1094 (87.5%) received at least one cephalosporin prescription, 799 (63.9%) received at least one macrolide prescription, and 559 (44.7%) received at least one other type of antibiotic prescription. However, frequent antibiotic use may cause MRSA occurrence. The prevalence of MRSA colonization in AD is approximately 10–30% and increases independently in areas worldwide^24,27,28^. Thus, careful use of antibiotics is necessary in AD patients. Penicillins or first-generation cephalosporins are used for methicillin-sensitive *S. aureus* (MSSA) infections, and doxycycline, clindamycin or sulfamethoxazole-trimethoprim have been recommended for MRSA infections^29,30^ by the Infectious Diseases Society of America. Therefore, further studies should also evaluate the influence of the above antibiotics on the development of new-onset asthma in AD patients.

The effect of antibiotics in preschoolers with asthma is much greater than that in older children as a part of our results. The supposed reasons are that the definition of asthma is different between preschool children and older children. The diagnosis of asthma in children older than 5 years depends on the Global Initiative for Asthma (GINA) guidelines^31^. However, there is no standard definition of asthma for preschool children that incorporates clinical symptoms, family history and drug response. In addition, the triggers of asthmatic episodes are also different between preschool children and older children. Viral or bacterial infection is an important etiology in preschool children, and the asthma of most preschool children goes into remission after they grow up. Thus, the association between antibiotic use and the development of asthma in patients with AD was easily observed in preschool children.

Our study had limitations in that we did not know the children’s parents’ socioeconomic status, the children’s exposure to secondhand smoke, or the history of atopic dermatitis in parents and siblings. Moreover, definitive diagnosis of asthma in preschool children is difficult, regardless of whether true asthma may have led to study inclusion.

Finally, we concluded that frequent use of antibiotics in AD patients might exacerbate the subsequent development of asthma. The antibiotic subtype, dose and duration of use should be taken into consideration when AD patients have infections.

## Methods

### Study design and data source

We conducted a retrospective nested case and control study designed to examine antibiotic exposure and risks of asthma development in children with atopic dermatitis. This study used the 2005 Longitudinal Health Insurance Database (LHID 2005), which provided the medical administrative claims of 1,000,000 random patients in the Taiwan National Health Insurance (NHI) program from January 1, 2005, to December 31, 2005. The NHI program covers approximately 25.68 million individuals, approximately 99.6% of the population in Taiwan. LHID 2005 includes information such as patients’ demographics; clinical diagnoses; medications; health-care utilization; and expenditure on hospitalization, clinics, ambulatory care, and home care. This study was approved by the Institutional Review Board of Kaohsiung Medical University Hospital.

### Study population

**Figure 2:**
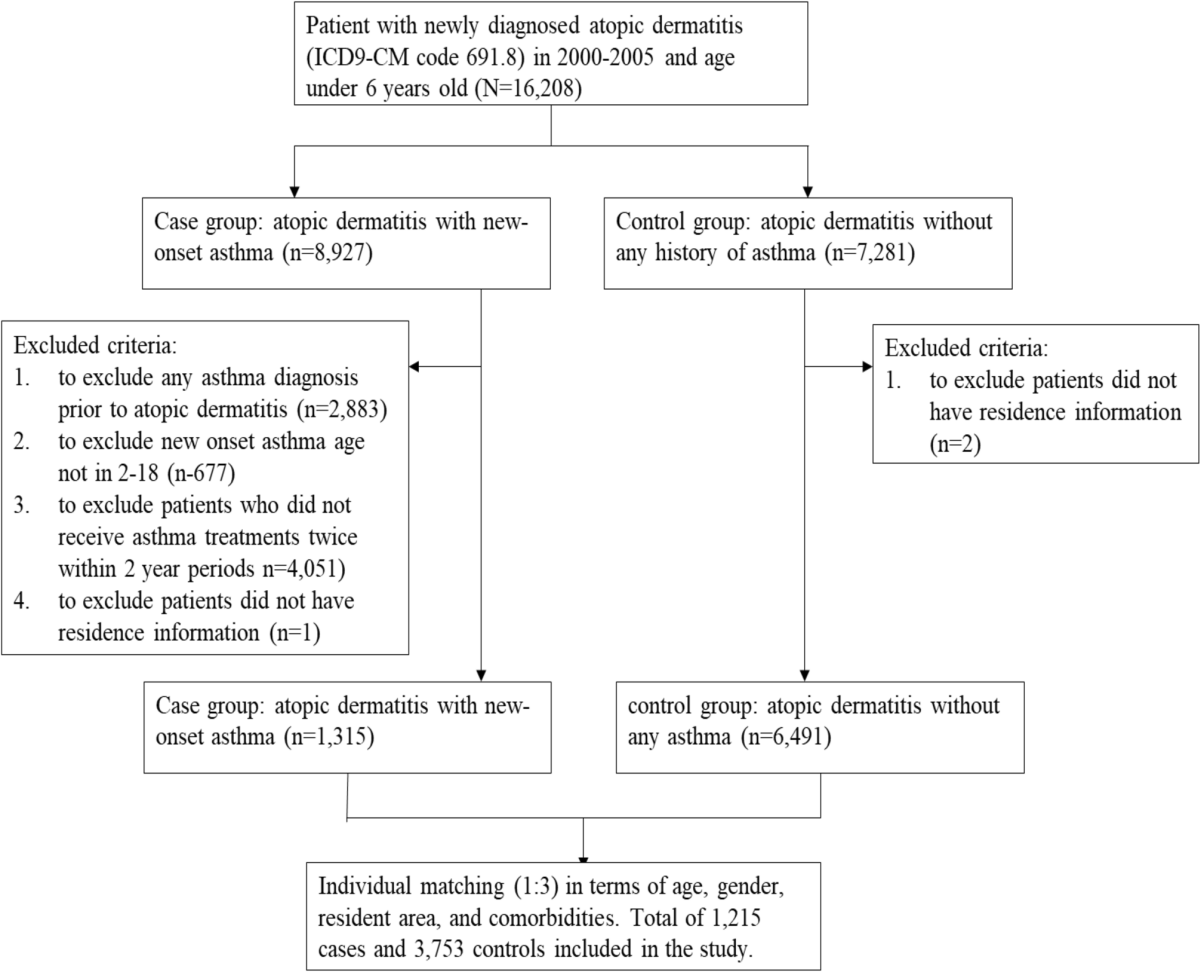
Flowchart of inclusion and exclusion criteria in this study.

By using the LHID 2005 database, we selected patients who were less than 6 years old with AD (ICD-9-CM code 691.8) who had never received antibiotic treatment before AD. The case group included AD patients with new-onset asthma, and the control group included AD patients without any medical history of asthma. Asthma diagnosis in Taiwan was made according to GINA guidelines^31^. Children younger than 5 years old were diagnosed with asthma based on respiratory symptoms, including coughing, wheezing, and fast breathing; parent atopic dermatitis history; and a decrease in symptoms after taking medication. New-onset asthma was defined as the first asthma diagnosis with ICD-9 CM code 493, combined with at least two prescriptions of anti-asthmatic drugs in different episodes during a 2-year period. Anti-asthmatic drugs include inhaled selective β2-agonists (Anatomical Therapeutic Chemical (ATC) code R03AC), combined inhaled salbutamol/sodium cromoglycate (ATC code: R03AK04), inhaled corticosteroids (ATC code: R03BA), and combined inhaled selective β2-agonists/corticosteroids (ATC code: R03AK06, R03AK07)^16^. The index date for the case group was defined as the date of first asthma diagnosis, and the index date was assigned to the same pairs of control children without asthma. Children who had an asthma diagnosis before AD or before age 2 years old were excluded to avoid any chronological overlap between exposure and outcome^16^. Because the baseline characteristics were significantly different between groups, which led to selection bias, we used a propensity score matching approach to match cases with comparable controls. The propensity score was generated in a logistic regression with the covariates shown in Table 1. Nonasthmatic control children were 3:1 matched to asthmatic children based on the propensity score. The flowchart of the inclusion and exclusion criteria is shown in Fig. 2.

### Exposure to antibiotics

Antibiotic medications were identified from the outpatient prescription claim database in LHID 2005. The outpatient prescription claims consisted of information including the dosage, date of prescription, duration, and total number of pills. Cumulative DDDs were calculated to investigate the effect of different antibiotic dosages. Antibiotic use was defined as the prescription of at least one antibiotic (ATC code: J01) within the 5 years prior to the index date. Penicillins (ATC code: J01C), cephalosporins (ATC code J01D), and macrolides (ATC code: J01F) were the three most common antibiotics used. Other antibiotic classes were also analyzed, including glycopeptides, lincosamides, sulfonamides, and quinolones. Based on the quartiles of the cumulative dosage distribution, cumulative DDDs were classified into different levels of dosage^17^.

### Potential confounders

In addition to age and sex covariates, following previous studies, we included potential confounders known to be related to asthma, including urban residence, allergic rhinitis (ICD9-CM code 477.8, 477.9), chronic rhinitis (ICD9-CM code 472.0), acute sinusitis (ICD9-CM code 461,473), and bronchiolitis (ICD-9 CM code 466.19)^17,18^. Confounders were identified from all claims made within one year before the index date.

### Statistical analysis

Pearson’s chi-square test was used to evaluate categorical variables between the case and control groups. The association between antibiotic exposure and the risk of asthma was analyzed by conditional logistic regression. Covariables and the type of antibiotic, including penicillins, cephalosporins, and macrolides, were adjusted by regression to evaluate the effect of antibiotic class. Odds ratios (ORs), adjusted ORs (aORs) and 95% confidence intervals (CIs) showed the risk of asthma development in antibiotic usage. The Hosmer–Lemeshow test was used to examine the goodness-of-fit of the model and p-value was 0.999, indicating models were well fitted. In addition, Cochran-Armitage trend test was used to test dose-dependency effects of the antibiotics use on risks of asthma. All statistical analyses were performed by SAS 9.4, and a p value below 0.05 was considered statistically significant.

## Supplementary Information

Supplementary Information.
