# Supplementary material for: The effects of antibiotic exposure on asthma in children with atopic dermatitis
Source: Sci Rep. 2021 Apr 19;11:8526. doi: 10.1038/s41598-021-87981-7 (PMC8055669; doi:10.1038/s41598-021-87981-7)
Supplement: Supplementary file 1 — Supplementary Information. [file 41598_2021_87981_MOESM1_ESM.doc]

**The effects of antibiotic exposure on asthma in children with atopic dermatitis**

I-Lun Chen, MD^1^#, PhD, Ming-Kai Tsai, MD^2^#, Hao-Wei Chung, MD^3^, Hui-Min Hsieh, PhD^4,5,6,7^, Yu-Ting Huang, MS^8^, Yi-Ching Lin, MD, PhD^5,9,10,11^*, Chih-Hsing Hung, MD, PhD^3,12,13,14,15^ *

^1^Department of Pediatrics, Kaohsiung Chang Gung Memorial Hospital, Chang Gung University, College of Medicine, Kaohsiung, Taiwan;

^2^Division of Nephrology, Department of internal Medicine, Kaohsiung Armed Forces General Hospital, Kaohsiung, Taiwan;

^3^Department of Pediatrics, Kaohsiung Medical University Hospital, Kaohsiung Medical University, Kaohsiung, Taiwan;

^4^Department of Public Health, Kaohsiung Medical University, Kaohsiung, Taiwan;

^5^Department of Medical Research, Kaohsiung Medical University Hospital, Kaohsiung Medical University, Kaohsiung, Taiwan;

^6^Department of Community Medicine, Kaohsiung Medical University Hospital, Kaohsiung, Taiwan;

^7^Center for Big Data Research, Kaohsiung Medical University, Kaohsiung, Taiwan;

^8^Division of Medical Statistics and Bioinformatics, Department of Medical Research, Kaohsiung Medical University Hospital, Kaohsiung Medical University, Kaohsiung, Taiwan;

^9^Department of Laboratory Medicine, Kaohsiung Medical University Hospital, Kaohsiung Medical University, Kaohsiung, Taiwan;

^10^Doctoral Degree Program of Toxicology, College of Pharmacy, Kaohsiung Medical University, Kaohsiung, Taiwan;

^11^Department of Laboratory Medicine, School of Medicine, College of Medicine, Kaohsiung Medical University, Kaohsiung, Taiwan;

^12^Research Center for Environmental Medicine, Kaohsiung Medical University, Kaohsiung, Taiwan;

^13^Department of Pediatrics, Faculty of Pediatrics, College of Medicine, Kaohsiung Medical University, Kaohsiung, Taiwan;

^14^Graduate Institute of Medicine, College of Medicine, Kaohsiung Medical University, Kaohsiung, Taiwan;

^15^Department of Pediatrics, Kaohsiung Municipal Siaogang Hospital, Kaohsiung, Taiwan.

#: The authors I-Lun Chen and Ming-Kai Tsai contributed equally to this work.

*: The authors Yi-Ching Lin and Chih-Hsing Hung contributed equally to this work.

***Correspondence and reprint requests to:**

Chih-Hsing Hung, MD. PhD.

e-mail: [pedhung@gmail.com](mailto:pedhung@gmail.com)

#100, Tz-You 1st Road, Kaohsiung 807, Taiwan, R.O.C.

Phone: 886-7-3121101; FAX: 886-7-3213931

Yi-Ching Lin, MD, PhD.

e-mail: winterjeanne@gmail.com

#100, Tz-You 1st Road, Kaohsiung 807, Taiwan, R.O.C.

Phone: 886-7-321101; FAX: 886-7-3213931

Appendix eTable 1. Classifications of antibiotic exposure within 5-year preceding index date and the risk of asthma development in children with atopic

|  | Case group | Control group | Adjusted model^a^ | | |
| --- | --- | --- | --- | --- | --- |
|  | Exposure number (%) | Exposure number (%) | aOR | 95%CI | P-value |
| Amoxicillin | 567 (45.3%) | 1,344 (35.8%) | 1.58 | 1.37-1.83 | <.0001 |
| Ampicillin | 28 (2.2%) | 58 (1.5%) | 1.46 | 0.89-2.37 | 0.1310 |
| Cefazolin Sodium | 180 (14.4%) | 381 (10.1%) | 1.50 | 1.22-1.84 | 0.0001 |
| Ceftriaxone Sodium | 2 (0.2%) | 5 (0.1%) | 1.37 | 0.20-9.61 | 0.7492 |
| Cephradine | 258 (20.6%) | 648 (17.3%) | 1.25 | 1.06-1.49 | 0.0097 |
| Cefaclor | 537 (42.9%) | 1,670 (44.5%) | 0.92 | 0.80-1.05 | 0.2211 |
| Ceftibuten | 6 (0.5%) | 10 (0.3%) | 2.02 | 0.65-6.28 | 0.2263 |
| Azithromycin | 235 (18.8%) | 271 (7.2%) | 3.44 | 2.76-4.29 | <.0001 |
| Clarithromycin | 28 (2.2%) | 40 (1.1%) | 2.87 | 1.63-5.04 | 0.0002 |
| Erythromycin | 3 (0.2%) | 16 (0.4%) | 0.52 | 0.14-1.85 | 0.3091 |
| **<5 years** |  |  |  |  |  |
| Amoxicillin | 334 (41.8%) | 777 (32.4%) | 1.56 | 1.30-1.88 | <.0001 |
| Ampicillin | 15 (1.9%) | 30 (1.2%) | 1.56 | 0.78-3.13 | 0.2106 |
| Cefazolin Sodium | 124 (15.5%) | 239 (10.0%) | 1.75 | 1.35-2.27 | <.0001 |
| Ceftriaxone Sodium | 1 (0.1%) | 4 (0.2%) | 0.58 | 0.04-9.84 | 0.7090 |
| Cephradine | 150 (18.8%) | 338 (14.1%) | 1.40 | 1.11-1.76 | 0.0038 |
| Cefaclor | 324 (40.5%) | 1,008 (42.0%) | 0.91 | 0.77-1.08 | 0.2857 |
| Ceftibuten | 2 (0.3%) | 7 (0.3%) | 1.01 | 0.18-5.76 | 0.9908 |
| Azithromycin | 130 (16.3%) | 141 (5.9%) | 3.80 | 2.80-5.18 | <.0001 |
| Clarithromycin | 11 (1.4%) | 15 (0.6%) | 2.66 | 1.10-6.42 | 0.0300 |
| Erythromycin | 3 (0.4%) | 9 (0.4%) | 0.88 | 0.22-3.56 | 0.8587 |
| **>5 years** |  |  |  |  |  |
| Amoxicillin | 233 (51.7%) | 567 (41.9%) | 1.61 | 1.27-2.04 | <.0001 |
| Ampicillin | 13 (2.9%) | 29 (2.1%) | 1.39 | 0.70-2.75 | 0.3492 |
| Cefazolin Sodium | 56 (12.4%) | 142 (10.5%) | 1.16 | 0.82-1.65 | 0.3987 |
| Ceftriaxone Sodium | 1 (0.2%) | 1 (0.1%) | 3.00 | 0.19-47.96 | 0.4373 |
| Cephradine | 108 (23.9%) | 310 (22.9%) | 1.09 | 0.84-1.42 | 0.4979 |
| Cefaclor | 213 (47.2%) | 663 (49.0%) | 0.93 | 0.74-1.17 | 0.5299 |
| Ceftibuten | 4 (0.9%) | 3 (0.2%) | 3.41 | 0.72-16.25 | 0.1237 |
| Azithromycin | 105 (23.3%) | 130 (9.6%) | 3.04 | 2.21-4.18 | <.0001 |
| Clarithromycin | 17 (3.8%) | 25 (1.8%) | 2.94 | 1.42-6.11 | 0.0039 |
| Erythromycin | (0.0%) | 7 (0.5%) | n.a. | - | - |

Note: aOR: adjusted odds ratios; CI: confidence intervals. a: conditional logistic regression models were used and adjusted for patients’ gender, age group, residential area and comorbidities (i.e., allergic rhinitis, chronic rhinitis, acute sinusitis and bronchiolitis) as listed in the Table1. P-values <0.05 were statistically significant.

Appendix eTable 2. Cumulative DDDs of antibiotics within 5-year preceding index date and the risk of asthma development in children with atopic dermatitis

|  |  | Case group | Control group | Adjusted model ^a^ | | | Cochran-Armitage Trend Test |
| --- | --- | --- | --- | --- | --- | --- | --- |
|  |  | Exposure number (%) | Exposure number (%) | aOR | 95%CI | P-value | P-value |
| **Any antibiotics** | No | 15(1.2%) | 154 (4.1%) | 1.00 |  |  |  |
|  | Yes | 1,236(98.8%) | 3,599 (95.9%) | 3.68 | 2.13-6.36 | <.0001 |  |
| Cumulative DDDs | no use | 15(1.2%) | 154 (4.1%) | 1.00 |  |  |  |
|  | <8.08 | 335(26.8%) | 1,174 (31.3%) | 3.16 | 1.81-5.51 | <.0001 | <0.0001 |
|  | 8.08-21.96 | 408(32.6%) | 1,115 (29.7%) | 4.25 | 2.42-7.45 | <.0001 |  |
|  | >21.96 | 493(39.4%) | 1,310 (34.9%) | 4.38 | 2.49-7.69 | <.0001 |  |
| **Penicillins** | No | 113(9.0%) | 543 (14.5%) | 1.00 |  |  |  |
|  | Yes | 1,138(91.0%) | 3,211 (85.5%) | 1.71 | 1.37-2.14 | <.0001 |  |
| Cumulative DDDs | no use | 113(9.0%) | 543 (14.5%) | 1.00 |  |  |  |
|  | <5.75 | 424(33.9%) | 1,317 (35.1%) | 1.52 | 1.20-1.93 | 0.0006 | <0.0001 |
|  | 5.75-14.75 | 351(28.1%) | 948 (25.2%) | 1.85 | 1.44-2.37 | <.0001 |  |
|  | >14.75 | 363(29.0%) | 946 (25.2%) | 1.96 | 1.52-2.53 | <.0001 |  |
| **Cephalosporins** | No | 157(12.5%) | 610 (16.3%) | 1.00 |  |  |  |
|  | Yes | 1,094(87.5%) | 3,143 (83.7%) | 1.36 | 1.11-1.66 | 0.0028 |  |
| Cumulative DDDs | no use | 157(12.5%) | 610 (16.3%) | 1.00 |  |  |  |
|  | <2.55 | 364(29.1%) | 1,115 (29.7%) | 1.29 | 1.03-1.61 | 0.0241 | <0.0001 |
|  | 2.55-6.68 | 304(24.3%) | 891 (23.7%) | 1.33 | 1.06-1.68 | 0.0146 |  |
|  | >6.68 | 426(34.1%) | 1,138 (30.3%) | 1.48 | 1.18-1.85 | 0.0008 |  |
| **Macrolides** | No | 452(36.1%) | 1,720 (45.8%) | 1.00 |  |  |  |
|  | Yes | 799(63.9%) | 2,033 (54.2%) | 1.51 | 1.31-1.75 | <.0001 |  |
| Cumulative DDDs | no use | 452(36.1%) | 1,720 (45.8%) | 1.00 |  |  |  |
|  | <1.50 | 366(29.3%) | 975 (26.0%) | 1.45 | 1.22-1.71 | <.0001 | <0.0001 |
|  | 1.50-4.29 | 232(18.5%) | 569 (15.1%) | 1.54 | 1.26-1.87 | <.0001 |  |
|  | >4.29 | 201(16.1%) | 490 (13.0%) | 1.65 | 1.34-2.05 | <.0001 |  |
| **Others** | No | 692(55.3%) | 2,675 (71.3%) | 1.00 |  |  |  |
|  | Yes | 559(44.7%) | 1,079 (28.7%) | 2.16 | 1.87-2.50 | <.0001 |  |
| Cumulative DDDs | no use | 693(55.4%) | 2,675 (71.3%) | 1.00 |  |  |  |
|  | <2.00 | 165(13.2%) | 396 (10.6%) | 1.77 | 1.43-2.20 | <.0001 | 0.0051 |
|  | 2.00-6.00 | 220(17.6%) | 447 (11.9%) | 2.00 | 1.64-2.43 | <.0001 |  |
|  | >6.00 | 173(13.8%) | 236 (6.3%) | 3.13 | 2.46-3.98 | <.0001 |  |

Note: aOR: adjusted; CI: confidence intervals. a: conditional logistic regression models were used adjusting covariates listed in the Table1 and p values <0.05 were statistically significant. Cochran-Armitage trend test was used to test dose-dependency effects of the antibiotics use on risks of asthma.
